# BioTapestry now provides a web application and improved drawing and layout tools

**DOI:** 10.12688/f1000research.7620.1

**Published:** 2016-01-08

**Authors:** Suzanne M. Paquette, Kalle Leinonen, William J.R. Longabaugh

**Affiliations:** 1 Institute for Systems Biology, Seattle, WA, 98109, USA

**Keywords:** Visualization, networks, open-source, gene regulatory networks, web applications, automatic network layout

## Abstract

Gene regulatory networks (GRNs) control embryonic development, and to understand this process in depth, researchers need to have a detailed understanding of both the network architecture and its dynamic evolution over time and space. Interactive visualization tools better enable researchers to conceptualize, understand, and share GRN models. BioTapestry is an established application designed to fill this role, and recent enhancements released in Versions 6 and 7 have targeted two major facets of the program. First, we introduced significant improvements for network drawing and automatic layout that have now made it much easier for the user to create larger, more organized network drawings. Second, we revised the program architecture so it could continue to support the current Java desktop Editor program, while introducing a new BioTapestry GRN Viewer that runs as a JavaScript web application in a browser. We have deployed a number of GRN models using this new web application. These improvements will ensure that BioTapestry remains viable as a research tool in the face of the continuing evolution of web technologies, and as our understanding of GRN models grows.

## Introduction

### Visualizing developmental GRNs

Gene regulatory networks (GRNs) are responsible for driving the process of embryonic development ^1^. This is an extremely complex process, and dedicated software tools are necessary to document both the network architecture and its dynamic evolution over time and space. Since a single static network figure does not adequately convey these complex behaviors, these tools also need to be highly interactive. The user should be able to explore the behavior of network subcircuits at particular points in time and space, and have access to relevant documentation of the underlying experimental evidence for each feature of the network. To be an effective and widely used tool, it must also be easy to share these interactive networks models over the web, rather than requiring users to download and install specialized software.

### Existing BioTapestry implementation

**Figure 1.**
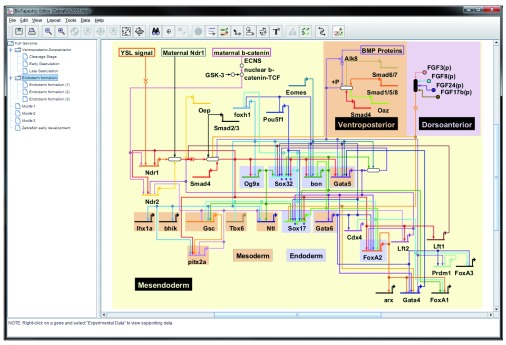
Screenshot of the BioTapestry Java desktop Editor. The early endoderm specification GRN for Zebrafish ^4^ as it appears in the BioTapestry Java desktop Editor.

**Figure 2.**
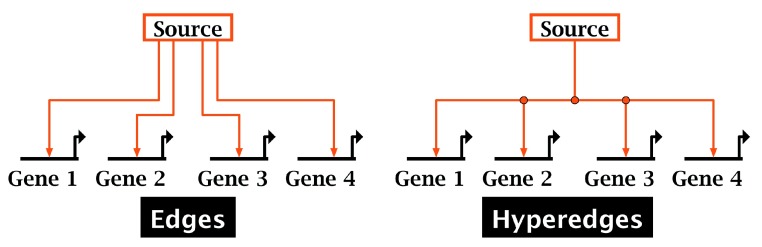
Edges and hyperedges. By sharing link segments, hyperedges (right) create a more parsimonious representation of a set of directed links than standard edges (left).

BioTapestry ^2, 3^ is an open-source software application that was developed to fill the need for a GRN modeling tool that can share interactive models over the web. Figure 1 shows the desktop BioTapestry Editor displaying a Zebrafish developmental GRN ^4^. BioTapestry has many notable features:

• It represents a complex network using a model hierarchy in which each child model is constrained to contain a subset of the network elements present in its parent model. This central feature allows researchers to organize and maintain models that track development of complex embryos over time and space, and is also useful for organizing any large network model.
• It represents the network with a level of abstraction that is appropriate for GRN models. General-purpose network visualization tools are not domain-specific enough to represent GRN clearly and effectively.
• It uses colored, orthogonal directed hyperedges, i.e. “circuit traces” or “link trees”; see Figure 2. These link trees provide a compact and unambiguous representation of the GRN edges.
• It allows users to associate experimental data or URLs with each network node or link.

### Recent improvements target two distinct areas

Recent BioTapestry development work, which has been released as Versions 6 and 7, focused on two distinct areas: new features to assist users to draw and layout networks, and a BioTapestry Viewer web application which runs entirely in a web browser.

***Bigger networks need better drawing support***. As noted above, BioTapestry represents the edges of the GRN using “link trees”. Experience working with users has demonstrated that it is helpful to provide tools that aid them in creating clean, well formed, unambiguous, and orthogonal link trees. It is also beneficial to provide automatic network layout tools, since BioTapestry has grown beyond its roots as a tool to draw small-to-medium sized networks, and can be used to visualize large networks as well.

***A new platform is needed***. BioTapestry has always been a web-centric application. Both the BioTapestry Editor (which is used to build the GRN model) and original Viewer (a read-only client for sharing the model on the web) were written in Java using the Java2D graphics library and deployed using Java Web Start (JWS). These two separate packages are the same program behind the scenes, with the Viewer exposing a limited subset of the functionality in the Editor. However, in recent years developers have been moving away from JWS as a platform for web-enabled applications. Increased emphasis on Java security has made it more difficult for users to quickly and easily launch a JWS application. In response, since September 2012 we have distributed the BioTapestry Editor as a downloadable executable that does not require JWS to run. This continues to support the BioTapestry Editor’s primary use as a GRN model-building tool, yet for simple and easy sharing of interactive GRN models on the web, we needed to consider an entirely new approach.

The first wave of migration away from Java-based browser tools for network visualization applications relied heavily on Adobe Flash. The Flash-based Cytoscape Web platform ^5^, STRING ^6^, and the myGRV component of myGRN ^7^ were examples of this trend. More recently, it has become possible to create rich 2D visualization web applications (software programs which run entirely within a web browser) using Scalable Vector Graphics (SVG) or HTML5 Canvas in combination with JavaScript, Hypertext Markup Language (HTML), and Cascading Style Sheets (CSS).

Existing JavaScript/HTML5/SVG visualization efforts include Google Charts, Protovis ^8^, and D3.js ^9^. The BioJS repository ^10^ is an example of a new resource that has been created to provide a framework for open-source browser components for biological data visualization. Two notable JavaScript libraries specifically targeted at network visualization are Cytoscape.js and Sigma.js. (Although D3.js can be used to visualize networks, it is not focused on that domain.) In particular, the current Version 3 of the Cytoscape Java desktop application ^11^ can export a set of files that can be hosted on a web server to present a network in the web browser using Cytoscape.js.

Given the growth of the JavaScript/HTML5/SVG visualization platform, graphics-rich biomedical web applications using these technologies have been appearing, such as Regulome Explorer and the Personal Genome Browser ^12^. These same technologies have also made it possible for us to now replace the JWS-based BioTapestry Viewer with a new web application.

## Methods

### Implementation

**Figure 3.**
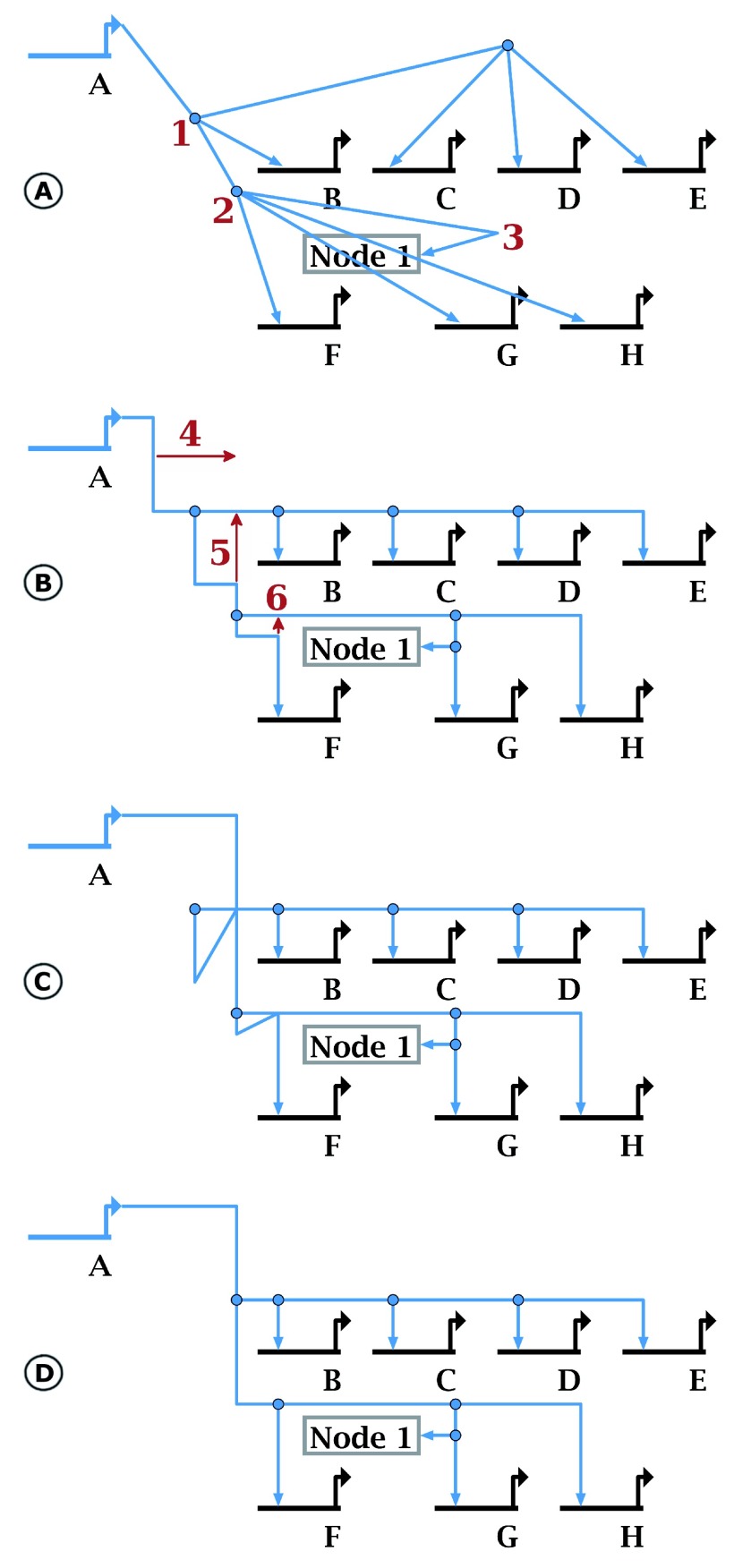
New drawing tools. The new orthogonalization tool (here we use **Layout->Fix All Non-Orthogonal Segments->Minimize Shifts**) takes existing “roughed-in” hyperedge geometry ( **A**) and tweaks it so it is orthogonal ( **B**). The resulting geometry is not generally optimal, since corner points (e.g. those labeled red **1** and **2** in ( **A**) are often retained. Gross features such as the 180-degree turn at red **3** must be provided to guide placement. In ( **C**), the user can quickly drag segments (red arrow **4**) and corner points (red arrows **5** and **6**). Although these changes create a severely malformed link tree, the overlap tool can repair it, making this approach a quick and easy shortcut for editing tree geometry. Selecting **Layout->Clean Up All Overlapping Link Tree Geometry** produces the clean result in ( **D**).

***Enhanced drawing support***. Our experience has shown that creating small tools to help users draw link trees can make network creation more efficient. Two recently introduced drawing tools are illustrated in Figure 3: one tool takes a tree that was drawn with diagonal links and tweaks it to make the segments orthogonal, and the second tool reorganizes the tree to eliminate ambiguous overlapping segments. The *orthogonality tool* allows the network creator to quickly “rough-in” the path geometry to approximate the desired final overall organization and let the system clean it up. Note that this tool does not do *de novo* layout of the links, but shifts and splits the existing link segments as needed to make links orthogonal. Since the tool rejects paths that overlap other links and nodes, and does not consider 180 degree turns in the possible solutions, the user may need to add additional link corners for guidance. The *overlap elimination tool* is designed to clean up incorrectly formed link trees, e.g. trees that have self-crossing or overlapping segments. The latter situation is particularly problematic, since the link tree may visually appear correct, but users clicking on the overlapping segments can get ambiguous results. One advantage of this tool is that users can create well-formed link trees by quickly dragging tree elements around to new, albeit incorrect, configurations and then have the tool clean up the mess, as shown in Figure 3.

Version 6 also introduced a new automatic layout technique, the *overlay-driven layout*. This technique leverages BioTapestry’s *Network Overlays*, which allow the user to create layers that annotate a GRN with *Network Modules.* Network Modules are collections of boxes that contain sets of related nodes. Users can also draw links between Network Modules to represent how the modules interact. In this fashion, the network creator can illustrate the abstract organization of the network at a high level.

**Figure 4.**
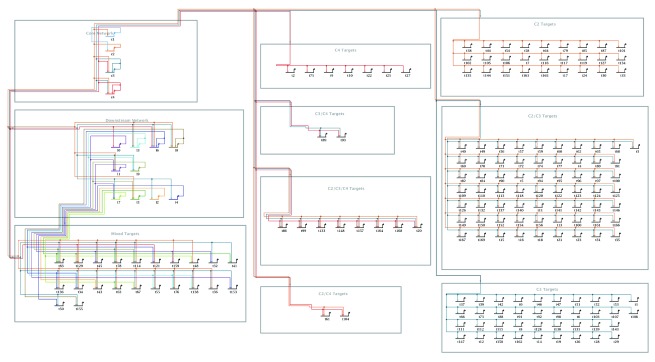
Example of overlay-driven strategy. This example is the end result of the overlay-driven layout use case described below. Using a simulated GRN data set (see Supplemental File 1 - https://f1000researchdata.s3.amazonaws.com/supplementary/7620/671ed832-fd5f-4130-a21e-c94b645b6720.sif), it groups genes into separate network modules. Two modules (upper and center left) contain “control genes”, while all other target genes are separated into modules based on their combination of inputs. Here the overlay intensity level is set near the minimum to de-emphasize the overlay features.

Merging the network overlay feature with BioTapestry’s existing automatic layout feature is a powerful combination, since the user can provide an overall automatic layout organization which is informed by their biological domain knowledge. GRNs can be broken down into biologically relevant sets of nodes, and the network layout will use these groupings to create a meaningful visualization. The layout also uses the module links from the overlay to route the edges between the nodes. An example is shown in Figure 4.

In order to create this feature, we added a new *stacked layout strategy* to the existing set of strategies that BioTapestry uses for automatic layout. Each BioTapestry layout strategy is designed to locate nodes and route the link trees in a fashion that avoids ambiguity and separates nodes and links in a rational fashion. For the stacked strategy, the goal was to create a simple rectangular organization containing rows of nodes. The difference between the stacked strategy and other traditional hierarchical layouts (e.g. the Sugiyama-styled layout ^13^) is that links are routed horizontally in reserved tracks above each node row, and traverse vertically between the rows in a shared, dedicated track down the left side of the block. While this canonical approach to link routing is far from the most parsimonious use of “link ink”, it is highly organized and utterly predictable. The stacked strategy also conforms to BioTapestry’s approach of treating genes as first-class citizens, with non-gene nodes preferentially grouped near the genes they are associated with. The network module groups in Figure 4 are all laid out using the stacked strategy.

In addition to its use as a building block in the overlay-driven layout, the stacked strategy works well for automatic layout of both selected subsets of nodes and single BioTapestry *regions*, so both of those new features were introduced in Version 6 as well. Regions are used in BioTapestry to represent different developmental domains, or more fundamentally, different regulatory states, in submodels of the model hierarchy. More details are available in 2, 3 and the BioTapestry Quickstart Tutorial.

***New architecture: A hybrid desktop/web design***. GRN models are most often publicly shared as a set of static images in a journal article, but the BioTapestry Viewer is intended to provide a richer and more interactive way of disseminating the GRN model if the creator chooses to do so. With an online model, users can explore the full model hierarchy, search for the sources and targets of genes, examine alternative paths between genes, and zoom in and out to more closely examine various aspects of the model’s architecture. An online model can also interactively provide experimental data and relevant citations. However, we still characterize the predominant use case for BioTapestry as a researcher using the full-featured BioTapestry Editor as a desktop Java application to create GRN models that are saved as local files on their computer. Given this situation, the redesigned BioTapestry needed to support the new read-only Viewer web application, while also continuing to support the existing Java desktop Editor. One approach is to have the Java application export the network as JavaScript Object Notation (JSON), and then create a standalone browser framework for rendering it; this is similar to the approach currently used by Cytoscape 3 and Cytoscape.js.

However, our development roadmap for the browser-based BioTapestry goes well beyond just viewing a completed, published network. Our ultimate goal is to support a full-featured browser-based Editor web application, enabling a distributed research community to collaborate on a shared GRN model using the browser-based tool. Thus, although our first step has been to create a browser-based Viewer, all the architectural decisions made were driven by this long-term goal of creating a browser-based BioTapestry Editor.

These requirements argued for a heavyweight server-side component driving the web application. We redesigned the BioTapestry architecture so the desktop Editor’s existing Java code base could also be hosted by a Java Servlet which supports a client interface running in an HTML5 web browser. This provides us with a migration pathway that continues to support existing users while allowing us to transition to a fully web-based user interface, all while maintaining as much of a common code base as is practical. The architecture we describe here has been used to produce the new Version 7 BioTapestry Viewer web application, and forms the basis for our work towards building a future BioTapestry Editor web application. This architecture can also serve as a roadmap for other development teams who are contemplating moving from Java Web Start to web applications.

***Issues addressed by the new architecture***

**Per-user application state**: Moving the code base from one that supported a single user on the desktop to multiple simultaneous users in a server required us to focus on separating out program state so that it could be stored in a per-session object. In the original single-user code, it was convenient to use Singleton objects ^14^, implemented as static class members, to provide globally accessible resources. However, we removed all these static variables, and now a separate per-session state object is maintained for each user; this session state argument was then added to many method signatures in the application. With the desktop Editor, a single instance of this state object is sufficient, but the web application creates, retrieves, and maintains a unique instance for each separate user session.

**Flow of control**: The original code base followed standard practice for writing an application built using Java Swing, where commands executed by selecting a menu item were written to extend Swing’s AbstractAction class, with the actionPerformed(ActionEvent e) method being overridden to implement the command. Whenever user input was needed to guide subsequent processing decisions, this method (or a subroutine) would make a call to display a modal dialog. The user’s inputs would become available for further processing once that dialog was dismissed. To move to an architecture where the server does not have to manage a separate thread on a per-session basis, we split these methods into a series of separate functions that maintain their state in the per-session state object. Each function in this series is called to update the current state based on user inputs, and return a request for any inputs needed by the next function in the chain. In the desktop case, these requests are still fulfilled by calling modal dialogs, but in the server case the thread launched for the request completes by returning the request for user information to the web browser.

As a result of this reorganization, the commands previously implemented as Swing AbstractActions are now implemented as ControlFlows, and we created two separate ControlFlowHarness implementations, which are frameworks to execute these commands from either a Swing desktop or web server context.

**Figure 5.**
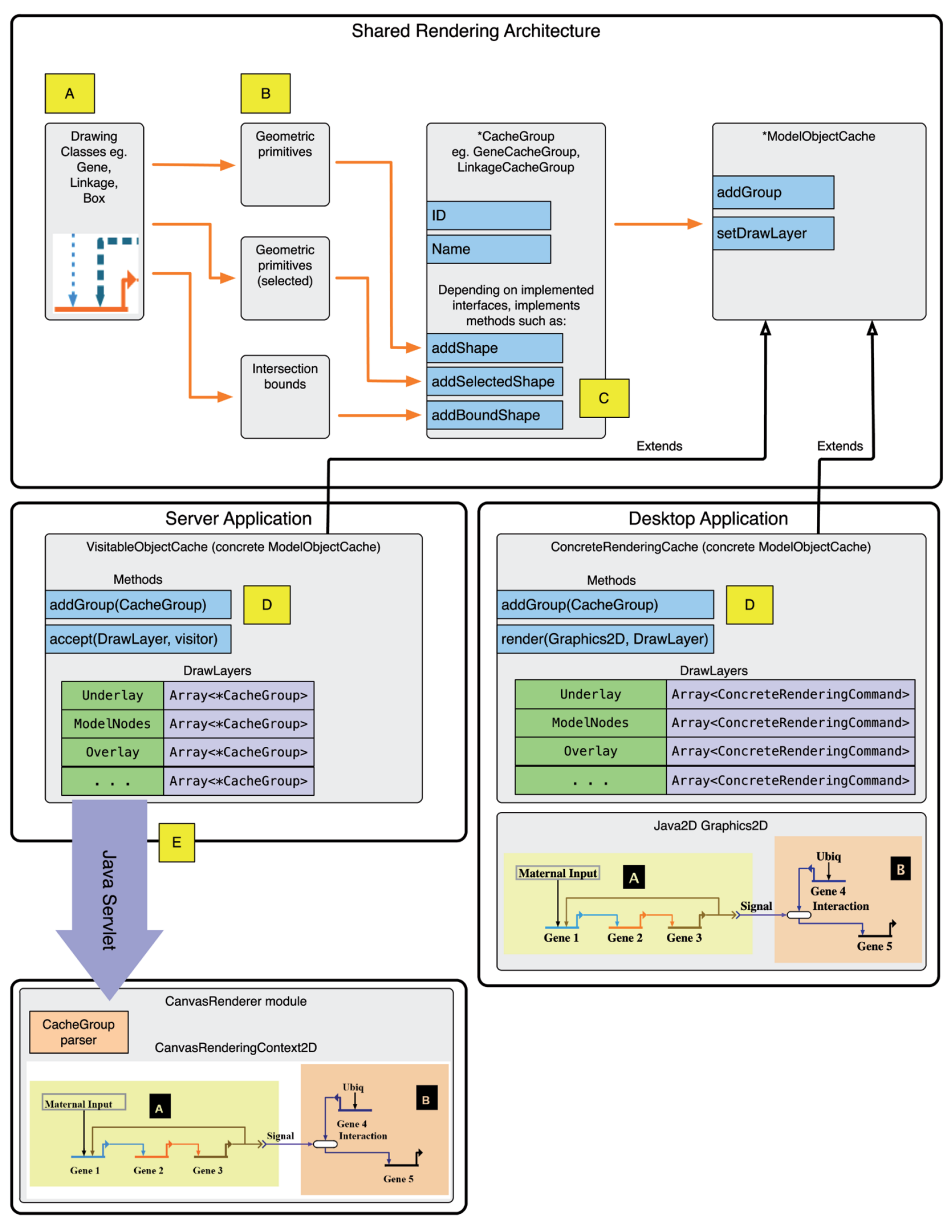
Architecture of dual-platform rendering pipeline. This is the architecture of the rendering pipeline. ( **A**): Each drawing class (e.g. a Gene) knows how to render the final glyph from primitive geometric shapes; additional shapes are also used to render the “selected” version. The classes also encapsulate information needed to perform intersection testing. ( **B**): In the desktop application, the logic for determining point intersections is provided by drawing class functions. In the server application, the rendering pipeline gets a list of intersection boundaries from each drawing class instance, and stores them in the CacheGroup. ( **C**): The concrete CacheGroup implementations have differing interfaces, depending on what needs to be exported to the web client while running as a server application. Shapes are added to the CacheGroups, which are then added to the ModelObjectCache; the concrete type of the ModelObjectCache depends on the target platform, as illustrated. ( **D**): In the server application, the addGroup() method of the VisitableObjectCache stores concrete CacheGroups to an array matching the appropriate DrawLayer. ( DrawLayers are used to organize the overall order of what is drawn first to last.) The CacheGroups are stored intact for serialization when the web client requests a model rendering update. The accept() method enables iterating through stored CacheGroups per DrawLayer. Alternately, in the desktop application, the addGroup() method of the ConcreteGraphicsCache extracts the primitive shapes from the CacheGroup and stores the shapes to an array matching the appropriate DrawLayer. The render() method then draws the stored shapes to a Graphics2D object given a DrawLayer. ( **E**): CacheGroup instances are not directly serialized for transmission. Instead, a helper class is used depending on the CacheGroup’s type; that is then serialized to JSON format.

**Dual rendering pipeline**: The rendering process had to be abstracted so that the same rendering code could either drive a Java2D rendering layer for the desktop application, or send a description over the wire to a remote client renderer. Originally, the rendering code drew directly to the screen using Java2D commands. The new Version 7 multi-renderer architecture now uses a layer of indirection, and all objects are rendered by first generating a stream of low-level geometric primitive shapes. These low-level shape streams can then be rendered on a variety of different platforms by simply implementing a thin platform-specific rendering layer for each platform. The architecture of our new split pipeline is shown in Figure 5.

In the new architecture, the primitives generated for a particular element such as a node are bundled into a CacheGroup object. For the desktop application, these CacheGroups are then used directly to drive the execution of Java2D rendering commands. Alternatively, for the web application, the CacheGroups are used by the model export logic to serialize the data into JSON, which is exported to the web browser for rendering. In addition to the rendering geometry, the exported CacheGroups also contain geometric primitives that, in combination with the network element ID stored in the CacheGroup, are used by the web client to perform mouse-click intersection testing that returns the element ID.

To reduce the latency of certain operations on the browser, such as clicking on a node so that it displays its orange “selected” highlighting, the server pre-generates certain rendering elements, such as all of these orange selected highlighting shapes, and ships these out in the CacheGroups along with the basic model geometry. The *network module* shapes that can be used in BioTapestry to annotate the network, which the user can toggle on and off, are also pre-generated for the web application. Both these pre-generating operations are only done for the web application; in the desktop, those shapes are only generated on the fly when they are needed.

For the web client, we needed to decide which technology to use for our rendering layer. As discussed above, HTML5 Canvas or SVG are the two main options for creating the rendering layer in the web browser, and we chose to use HTML5 Canvas. We based our decision on performance concerns (the Cytoscape.js project reported they decided to move from using SVG to HTML5 Canvas for performance reasons), and on the high degree of similarity between the HTML5 Canvas Application Programming Interface (API) and the Java2D API we already are using in BioTapestry.

We did encounter some issues while using Canvas. Perhaps the most onerous were font size inconsistencies. Certain nodes (e.g. box nodes) have their size set using the size of the enclosed text, and this calculation occurs on the server. Yet the same point size and font family produces a string of different dimensions on the server and different browser types; even the server operating system was a factor in this mix. We needed to implement logic in the web client renderer to use the HTML5 Canvas text metrics facility to calculate the required affine transform to match the text token dimensions provided by the server. Other problems we encountered were with dashed link rendering, since Internet Explorer 9 does not provide the API for setting dashed line rendering; we needed to implement a workaround using bitmaps. We also encountered some differences in compositing operators, since HTML5 Canvas implementations do not currently support a “clear” rule.

**Cross-platform specification classes**: The original desktop application created user interface (UI) elements, e.g. menus and toolbars, directly using available Swing components. To support a web application deployment, we created abstract, cross-platform descriptors to specify the contents of UI elements. These descriptors can then be used to create a Swing component for the desktop, or be serialized to JSON and sent to the browser, where a corresponding set of JavaScript UI components is created.

**Cross-platform dialog factories**: In the new architecture, dialogs implement a common interface that the web and desktop clients interact with. They are generated by Factory classes which return instances appropriate for the deployment context. On the desktop, the Factory returns a Swing dialog, while the web application context provides the information needed by the web client to construct the dialog, including a description of any information, including user inputs, the web application needs to collect and return to the server. This design approach allows future dialog modifications to take place in a single Java source code file and helps to prevent the divergence of the web and desktop clients.

***Web client implementation***

**Figure 6.**
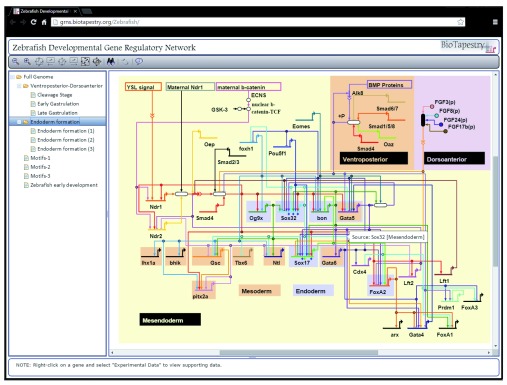
Screenshot of the BioTapestry Viewer web client. The early endoderm specification GRN for Zebrafish ^4^ as it appears in Google Chrome v47, Windows 7, served by Apache Tomcat version 7.0.55. Compare to desktop Editor version in Figure 1.

**Choosing Dojo**: To build the BioTapestry web client, we needed a JavaScript framework that had API documentation that was consistently available and kept up to date, an active community addressing bugs and producing new features, and a quick development time. To inform our decision of which framework to use, we built small BioTapestry prototypes in Ember.js, AngularJS, and the Dojo Toolkit. Based on our criteria and the experience of building these prototypes, including time to learn the framework’s API, the Dojo Toolkit covered all of our needs. It comes with a large library of user interface widgets that incorporate accessibility standards and manage browser/OS differences via a single API, all of which can be extended to perform as required. Dojo modules can also be loaded asynchronously on an as-needed basis [Asynchronous Module Definition (AMD)]. This ensures the web application will only use those network and client resources that are required as defined by how the user makes use of the application. The result is shown in Figure 6; compare this representation to the Java desktop Editor version in Figure 1.

**Virtual scrolling system**: Users can pan, scroll, and zoom GRN models in the network view panel. With the Java2D desktop implementation there is no severe performance penalty for using a JPanel matching the size of the whole model at the specified zoom level, which can often be quite large. However, this becomes a problem in the web client, because Canvas elements in the browser use a significant amount of memory at larger sizes, enough that a very large Canvas element can cause the browser to malfunction or crash. To address this problem, in the web client we implemented a ‘virtual’ scrolling and zooming system that restricts the Canvas element’s size to the drawing viewport’s display area. Panning, zooming, and scrolling events are handled as drawing functions, rather than using the native browser functions. This keeps the Canvas using the minimum amount of memory required while providing the same zooming, scrolling, and panning functionality users would expect.

## Operation

### Requirements

The Java Runtime Environment (JRE) Version 1.5 or newer is required to use the Java desktop Editor. JRE 1.5 and Apache Tomcat Version 6 or 7 are required to host a model on a web server. Chrome 4.0+, Firefox 3.5+, Safari 4.0+, and IE 9.0+ with JavaScript enabled are required to use the web application client. Any operating system that supports these technologies can be used to host a model, view it in the web client, or run the desktop Editor.

### Workflow: Creating a model using the BioTapestry desktop Editor

Because we redesigned BioTapestry in Version 7 in a manner that allows the traditional desktop Editor to continue to operate as it always has, there is no change to the way a user would create a GRN model using BioTapestry. The online “Quickstart” tutorial is still the best way to learn how to use the tool, and existing additional online documentation shows how to use other more advanced features.

### Workflow: Deploying a model using the BioTapestry web Viewer

Sharing a GRN model over the web using the new Viewer web application is significantly different than previous methods. The BioTapestry Viewer web client is distributed as a Java Web Application Archive (WAR) file that contains the BioTapestry Version 7 Viewer clients in both Java and JavaScript, and all required libraries (flexjson 2.1, the Dojo Toolkit 1.10, underscore.js, put-selector, dgrid, and xstyle). The WAR file can be placed in an active Apache Tomcat Web Application deployment directory (the server default is $CATALINA_HOME/webapps/), and by default Tomcat will automatically unpack the WAR file and deploy the application into a folder that matches the WAR file’s name. This will also dictate the URL at which the BioTapestry web application is available. For example, on a locally running instance of Apache Tomcat, which is accessible on port 8080 by default, BioTapestry.war will deploy to http://localhost:8080/BioTapestry/.

### Workflow: Changing GRN model files

The base BioTapestry web application distribution comes with a plain GRN model for testing the application’s deployment. There are two methods for loading a new GRN model file into your deployment: editing the already deployed application directly, or, editing the contents of the WAR file and (re)deploying it. For an active BioTapestry web application, place the new GRN model file into the WEB-INF/data/ folder of the deployed web application and edit the modelfile entry of the configuration.txt file to reflect the name of the new file, then reload the web application from the Apache Tomcat Web Application Manager. To edit the BioTapestry web application WAR file itself, use Oracle’s jar utility, or a compression utility capable of working with ZIP format archives. Make the same changes as you would to a live deployed BioTapestry web application, and then redeploy the WAR file.

Detailed instructions for working with the web application, including installing and customizing it, are available from the project web site.

## Use cases

### Using the network overlay-driven layout

**Figure 7.**
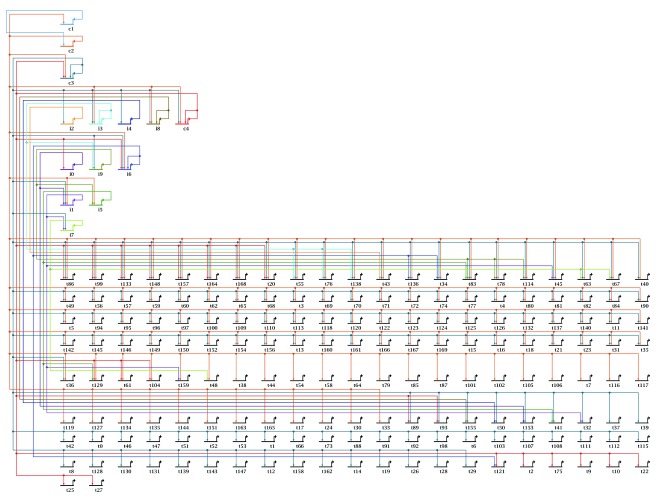
Overlay-driven layout: Initial input. Importing a network from a tab-delimited .sif file, and using the stacked automatic layout strategy, produces the result shown here. This shows how this new layout strategy can be applied to the whole network.

**Figure 8.**
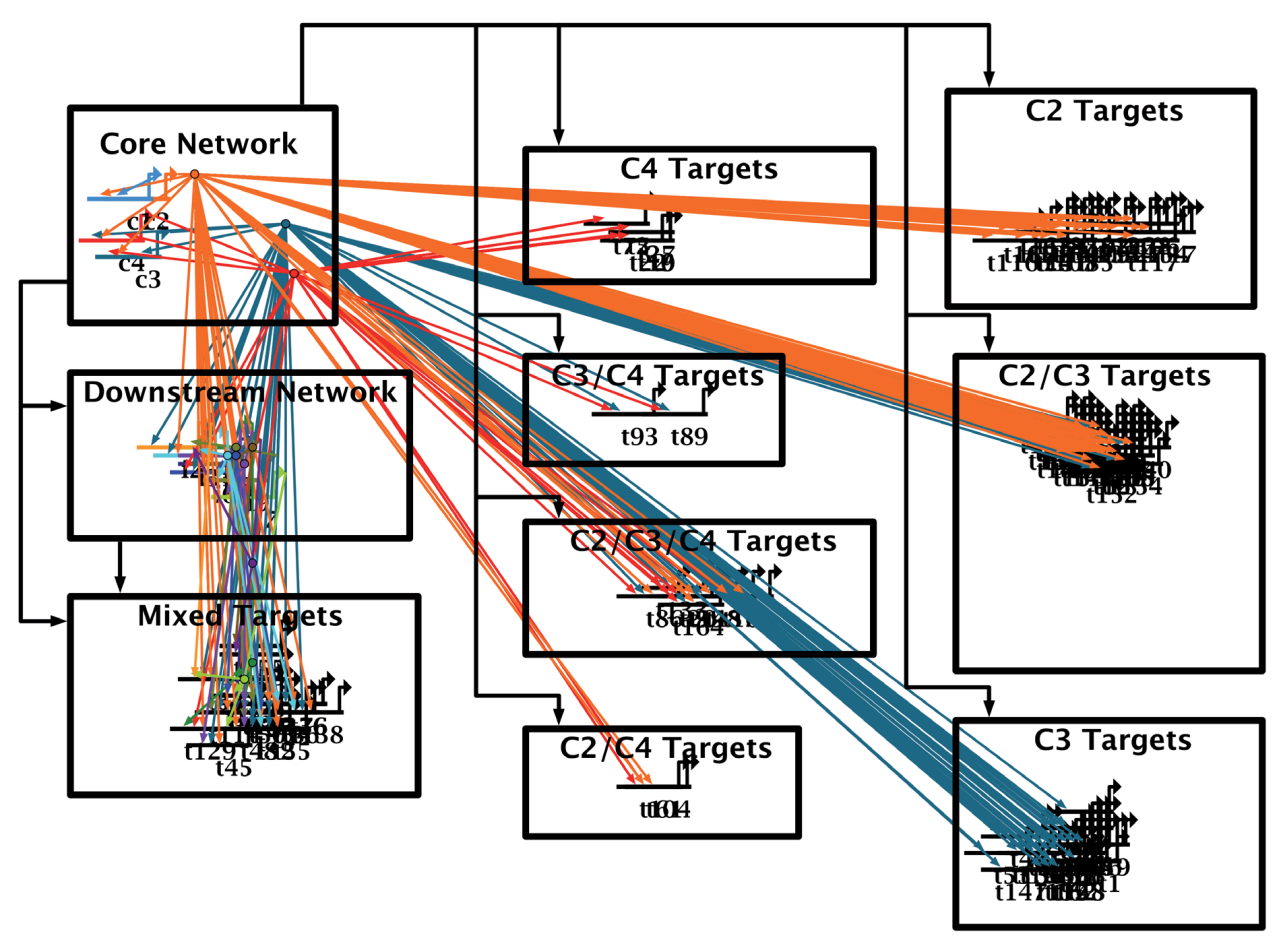
Overlay-driven layout: Preparing for layout. After the user creates a network overlay, and drags genes into piles, they draw a network module around each. This action automatically adds the genes to the modules. Then, after moving each module to the desired location, the user draws directed network module links between interacting modules. The next step will be to apply the overlay-driven layout algorithm.

As an example of how to use the overlay-driven layout, we want to create a layout where the “control” genes with outputs are broken out separately, and “target” genes with only inputs are grouped into blocks based upon common sets of inputs. This allows us to clearly see the control circuitry, while providing a useful way to break the large number of targets into similar groups. To do this:

1) Import a tab-delimited Cytoscape .sif file using BioTapestry’s **File->Import->Import Root Network From SIF...** command; this particular example uses a simulated GRN data set (see Supplemental File 1 - https://f1000researchdata.s3.amazonaws.com/supplementary/7620/671ed832-fd5f-4130-a21e-c94b645b6720.sif). Using a stacked strategy, keep the defaults except for setting the maximum row size to *20* and the target grouping strategy to *Order Targets by Source*. The resulting network is shown in Figure 7, and illustrates how the new stacked strategy organizes a full network.
2) Using the command **Edit->Manage Network Overlays and Modules->Add Network Overlay…**, create an overlay with a presentation style of *Transparent* and named e.g. “Target Groups”.
3) Drag the genes that will go into different modules around so each set can easily be contained in a bounding box. Then, using **Edit->Manage Network Overlays and Modules->Draw a New Network Module…**, draw a *One Box* type module around each group of nodes (the other module types do not support the overlay-driven layout). Modules boxes are drawn by clicking to start the box, and then clicking to end it.
4) Drag each module to the desired location by right-clicking on the module name or boundary and selecting **Move Module**. With the *One Box* type module, dragging the module name or the genes to reposition them will cause the module boundaries to expand if needed to still enclose all module contents. For best auto-layout results if the module boundaries have been automatically expanded in this way, right-click on the module name or boundary and select **Resize Core Module Definition to Current Visible Bounds** if that option is enabled.
5) Using **Edit->Manage Network Overlays and Modules->Draw a New Network Module Link…**, draw orthogonal *Promote* module links trees between all the modules that have interactions. See Figure 8. (Note: the link trees here have been reduced from the original arrangement in Figure 7 for clarity, but this is not necessary.)
6) Select **Layout->Apply Auto Layouts->Per Current Overlay**. If there are problems with the overlay (e.g. a required link between interacting modules is missing, or a module link is crooked), an error dialog will appear; these problems must be fixed before trying again. When the layout is complete, the default settings create the final result that was shown above in Figure 4. Figure 4 has the overlay *Intensity* level dialed down to near the minimum to better show the inter-module links.
  Note that the module boxes are enlarged as needed to contain the stacked layouts, since the system tries to maintain the relative horizontal and vertical orderings of the module features (i.e. edges and link positions). Thus, some module boxes may become larger than necessary, and manual tweaking may improve the aesthetics. It also helps to draw the original overlay with this ordering in mind. Finally, the modules are not resized to best position the module label, which often needs to be relocated for best results.

### Models deployed using the new BioTapestry Viewer

***MTB Network Portal***. The first deployment of the BioTapestry Viewer web application was an interactive network model for the Mycobacterium tuberculosis (MTB) Network Portal ^15^. This portal provides resources for computational modeling of host/pathogen interactions in *Mycobacterium tuberculosis*, and the BioTapestry web client handles the Environment-specific Gene Regulatory Network. This deployment of the web client needed to support experimental data hosted on external servers and sourced from web links embedded in the BioTapestry file. Thanks to BioTapestry’s implementation of an experimental data display plugin API, we were able to produce a custom Java plugin to retrieve this data and display it when users open experimental data pages.

***Ectoderm/Endomesoderm Networks***. The BioTapestry Java Web Start Viewer was originally developed to host an interactive model of the GRN controlling the first 30 hours of development of the endomesoderm in *Strongylocentrotus purpuratus* ^16^. This model has now been completely ported to the new Viewer platform. Additionally, the recently added GRN model for ectoderm development, based upon 17– 19, is hosted using our new BioTapestry Viewer as well.

***New BioTapestry model repository***. In order to provide access to other GRN models produced with BioTapestry using the new Viewer platform, and to provide a hosting site for models that were previously available only via Java Web Start, we have created a model repository. This site hosts some previously published models ^4, 19– 25^, including some models that were “orphaned” when their original websites were shut down. It is our hope that this repository will continue to grow and serve as a reliable method for accessing GRN models via a web browser. The site provides a “Quickstart” user guide, complete with screenshots, to orient new users to the BioTapestry web client's functionality.

## Conclusions

As GRN models continue to grow larger and more complex, we will continue to add features to BioTapestry that will aid researchers in building them. The layout tools we have described here are the most recent examples of such improvements. Our new BioTapestry Viewer web application ensures that users will continue to have access to dynamic, interactive GRN models online. Since this new architecture provides a richer server/browser interaction that only downloads data to the client when it is requested, we expect the new system to be better at handling very large and complex model hierarchies as GRN models grow in the future.

We are continuing to refactor the Java desktop Editor to use the new architecture, and plan to implement a web application of the Editor that can be used by research communities to collaborate on GRN models online. Given the cross-platform nature of our design, we will be able to do this while continuing to fully support the BioTapestry Java desktop Editor. Our GRN website will foster community involvement with GRN models as we continue to build it out. The site’s current compliment of models has amply demonstrated how the new web application Viewer can support interactive exploration of GRN models.

## Software availability

### Software available from

https://github.com/BioTapestry

### Archived code at the time of publication

http://dx.doi.org/10.5281/zenodo.35447 ^26^

http://dx.doi.org/10.5281/zenodo.35664 ^27^

### Licensing

BioTapestry source code (Java, JavaScript, HTML, CSS): GNU Lesser General Public License (LGPL) V 2.1. Some of the toolbar image files are freely distributed under a separate license from Sun Microsystems, now Oracle. Other libraries are also used in the server and client. The Dojo Toolkit, dgrid, xstyle, and put-selector are distributed under a Modified Berkeley Software Distribution (BSD) License. Flexjson is distributed under Apache License Version 2.0, and underscore.js is distributed under an MIT License.

## Supplementary materials

**Supplemental File 1.**

This is the mockup GRN definition file referenced in the overlay-driven use case. The file is in tab-delimited Cytoscape Simple Interaction Format (SIF), i.e. of the form source node **<tab>** interaction label **<tab>** target node. Networks in this format can be imported into a number of network visualization programs, such as Cytoscape, BioTapestry, and BioFabric.
