# Supplementary material for: BioTapestry now provides a web application and improved drawing and layout tools
Source: F1000Res. 2016 Jan 8;5:39. [Version 1] doi: 10.12688/f1000research.7620.1 (PMC4841208; doi:10.12688/f1000research.7620.1)
Supplement: Peer review history

# Peer review: Referee response for version 1

**DOI:** 10.5256/f1000research.8206.r12952

**Published:** 2016-04-01

**Authors:** Morris Maduro

**Affiliations:** 1 Department of Biology, College of Natural and Agricultural Sciences, University of California Riverside, Riverside, CA, USA

Gene Regulatory Networks describe the causal relationships, in space and time, among transcription factors and signaling events that drive processes under genetic control. The most frequent application of GRNs is to describe developmental processes, such as the specification of germ layer fates in the sea urchin embryo.

This article describes the general functionality of BioTapestry, in use since 2005, and its usefulness in describing the dynamic architecture of gene regulatory networks. In particular, improvements the tools used for drawing the connectivity in a network are described. The drawing tools are useful and intuitive and the authors justify the use of a "stacked layout strategy" to break down a large network into sets of functionally important nodes. Changes to the software are detailed as they apply to versions 6 and 7 of BioTapestry. Improvements include updating the software to run in a browser. For specific details about usage and installation, users are referred to online tutorials. By way of example, instructions are included for using the 'network overlay-driven' layout.

Migrating to a newer, interactive web-based platform presents itself with a number of logistical issues which are described in the article in detail, with a view toward eventually migrating BioTapestry to a web-based, collaborative platform. Logistical issues include the separation of functions to the server or the user's desktop, and cross-platform consistency in font and line rendering.

The text itself is well-written, and contains active links to many applications in context.

Comments:

1. The simulated GRN set is useful for demonstrating the modeling diagrams, but it could be made much simpler if it had fewer downstream targets, i.e. to simplify Figs. 4, 7, 8.
2. Some parts have a bit too much detail regarding how the authors dealt with specific problems, but these are not too distracting.
3. The Microsoft browser is now Edge. Edge seems to support the web-based viewer for the networks available via http://grns.biotapestry.org. This could be indicated in the first paragraph of the 'Operation' section.
4. As a final comment, while the authors consider evolving web technologies, they may also consider creating an "App" that can be installed on tablets and portable devices. This might be easiest to develop for the Android platform as this uses Java.

The authors have presented a very nice article that informs readers of updates to BioTapestry, which continues to be a terrific tool for visualization of gene network interactions.

I have read this submission. I believe that I have an appropriate level of expertise to confirm that it is of an acceptable scientific standard.

# Peer review: Referee response for version 1

**DOI:** 10.5256/f1000research.8206.r12950

**Published:** 2016-03-29

**Authors:** Tanja Muetze

**Affiliations:** 1 Imperial College London, London, UK

The authors present version 6 and 7 of BioTapestry in which they introduced improved network layout and drawing capabilities and added an in-browser JavaScript web editor functionality to BioTapestry as a hybrid between desktop and web app. BioTapestry aims to visualize and analyze gene regulatory networks and make it easy for users to share them.

The article is clearly written and has a good flow, despite being occasionally a bit informal. The article follows a clear structure with a descriptive title as well as a well-described abstract and conclusion that summarize the work. It links to documentation/tutorials as well as the code.

As another referee noted, the authors followed a recent trend for more web security by using a HTML Canvas, CSS and JavaScript approach instead of deploying the app via Java Web Start which runs Java code as previously done, a design decision that I support.

The following suggestions could further improve the article.

1. The proposed software was created about two years ago. I would encourage a timelier publication of this article to promote awareness of the software as well as to be a resource and reference guide for users.
2. On similar terms, the Github code repository seems to be a bit outdated. The last commit was 2 years ago. Consider how version updates of dependent software might impact BioTapestry despite version backward compatibility. Currently, Java 8 is distributed while this update to BioTapestry was initially written for Java 5.
3. Integration with other (network) tools, such as Gephi, would be desirable. The website mentions that export to SBML and SIF (for Cytoscape) are currently supported. It might be of interest to open a voting on the website (e.g. based on IP address and/or browser session to discourage multiple votes) to find out which import/export capabilities are most desired by most users. This would provide a fluent integration of BioTapestry with other bioinformatics, network or general software and would be an incentive for users of other programs to start using BioTapestry.
4. More on a side note, while being very descriptive and conversational, the article could have been more concise and separated future development from current design choices.

 

Overall, the authors did a very good job at developing and clearly presenting an update to BioTapestry, an important tool for gene regulatory network analysis and distribution.

I have read this submission. I believe that I have an appropriate level of expertise to confirm that it is of an acceptable scientific standard.

# Peer review: Referee response for version 1

**DOI:** 10.5256/f1000research.8206.r11875

**Published:** 2016-01-18

**Authors:** Matt Loose

**Affiliations:** 1 School of Life Sciences, University of Nottingham, Nottingham, UK

BioTapestry is the pre-eminent viewer for building and analysing developmental gene regulatory networks. Although many other GRN viewers are available, very few provide the ability to link developmental processes and the concepts of tissues and cell types emerging over time with the underlying GRN. BioTapestry was the first to really formalise this process (I believe building on the work of Schilstra and colleagues with NetBuilder) and provide a method of displaying GRNs incorporating the logic first proposed by Eric Davidson. The ongoing development and maintenance of tools like BioTapestry is very important to the understanding of GRNs in development. 

This manuscript is clearly written, accurately described and focuses on key improvements made in BioTapestry versions 6 and 7. I approve of the migration to a more web centric view for BioTapestry future development and look forward to the ability to edit GRNs in a web based environment. The focus on layout tools (the 'hyper edges' are an example) is extremely useful for cleaning up a visualisation. The network overlay-driven layout features seem to work well for clustering groups of genes in perhaps biologically meaningful ways.

The option to right click on a network link to visualise all the data behind that particular interaction is very helpful indeed.

The source code for BioTapestry is appropriately available from github and the documentation accompanying its release is clear and easy to follow.

One possible problem with BioTapestry is with respect to its deployment. The BioTapestry target audience is "...a researcher using the full-featured BioTapestry Editor as a desktop Java application to create GRN models that are saved as local files on their computer." (Page 6, Paragraph 1). This user will also tend to be a wet lab experimentalist collating data from direct observation as well as pooling other sources of knowledge, be that from the literature or - perhaps - other bioinformatics pipelines. The process by which such an individual shares their network becomes complex. The user is expected to set up an Apache Tomcat installation and configure the package. At the same time, the authors present a model repository (Page 12, Para 3). These concepts should perhaps be linked. It would be great if users could submit models quickly and easily to the model repository which - itself - could be searched by others or a direct link to it could be shared with others. Of course, it may not be a single experimentalist working on the model either - perhaps there is a larger team at work who all wish to work on the model at once.

Perhaps a hinderance to this process at the moment is that it appears each model visualised via the web viewer requires its own full install of the BioTapestry WAR file. A possible future improvement to the deployment method might be to pass the appropriate configuration files to the BioTapestry package at launch. This could then simplify the process of hosting a GRN model server visualised in BioTapestry.

A minor comment that might improve usability is with respect to the search features for nodes(genes) of interest. The default behaviour currently is "Match Full Name". This usually failed for me when browsing the endomesoderm network (as an example) whereas switching to "Match part of name" found the required gene or pathway (nice that it highlights pathway members in the case of signalling molecules!) straight away. An auto suggest function or simply switching the viewer to defaulting to "Match part of name" would increase usability.

A final broader comment with respect to the viewer. As far as I can see there is no way to export a model file (either .sif or any other format) from a network model. Whilst BioTapestry is the clearest and easiest way to draw developmental GRNs, many other tools exist for the analysis of networks. To have an easy way to export a collection of interactions from a BioTapestry web view would be helpful. This would enable users to analyse a network they were viewing in other tools if they so wished without having to redraw or otherwise reconstruct the network.

Overall I thank the authors for their continued work on BioTapestry - a tool which is incredibly useful for all those interested in the study of developmental GRNs.

I have read this submission. I believe that I have an appropriate level of expertise to confirm that it is of an acceptable scientific standard.

# Response

**Published:** 2016-01-21

**Authors:** William Longabaugh

**Affiliations:** Institute for Systems Biology, Seattle, USA

Dear Prof. Loose:

Thank you very much for taking the time to review our paper. I believe that your review contains four excellent points about current BioTapestry shortcomings, to wit:

1) Current model deployment is too complex:

We agree completely that the requirements for publishing an online model need to be greatly simplified. Our new model repository is a small, first step in this direction. Our current BioTapestry development proposal (for post Version 8, which is scheduled for 2016 release) is to significantly enhance grns.BioTapestry.org to allow models to be easily uploaded, searched, and shared. Combined with the online editor we are currently developing, it will make collaboration by distributed groups of researchers possible.

2) Current requirement for full WAR install:

The current requirement of running a separate servlet for each model is indeed far too restrictive. Your suggestion to make multiple models available from a single WAR deployment is spot on. This is also on our development roadmap, and is dependent on us completing a detailed thread-safety analysis followed by refactoring of the main control flow to improve parallelism of the servlet. Running multiple servlets is currently our workaround to improve overall concurrency.

3) "Match full name" as current search default:

Absolutely true; thanks for pointing this out. We plan to include this suggestion in our upcoming 7.0.1 bug-fix release.

4) No way to export network as, e.g., a SIF file:

The lack of a download/export option in Version 7 can trace its roots back to the original no-permissions, security-sandboxed Java WebStart implementation. You are absolutely correct that since we are now operating in a browser, those restrictions are removed, and we need to open up our thinking! It should be possible to provide this feature in the upcoming Version 8. But this feature is likely to be specified on a per-model basis, since we have found some labs have preferred to provide the underlying model files through more traditional one-on-one email correspondence.

Finally, though this paper did not dwell on the origins of BioTapestry [Longabaugh, Davidson & Bolouri, Dev Biol. 283(1), 2005], you are absolutely correct that it was a follow-on to the original NetBuilder project [Schilstra, M.J., Bolouri, H.: Logical Modeling of Developmental Genetic Regulatory Networks with NetBuilder [abstract]. In: Proceedings of the Second International Conference on Systems Biology (ICSB2001); 2001 Nov 4-7, Pasadena CA, abstract 112. Available at http://icsb2001.net/Posters/112_schilstra.pdf; current project home page is http://homepages.stca.herts.ac.uk/~erdqmjs/NetBuilder%20home/NetBuilder/]. Thanks for pointing that out.

Bill Longabaugh
